# Detection of *Streptococcus agalactiae* colonization in pregnant women by using combined swab cultures: cross-sectional prevalence study

**DOI:** 10.1590/S1516-31802010000200003

**Published:** 2010-03-04

**Authors:** Camila Marconi, Talita Trevizani Rocchetti, Vera Lúcia Mores Rall, Lidia Raquel de Carvalho, Vera Terezinha Medeiros Borges, Márcia Guimarães da Silva

**Affiliations:** I Undergraduate student, Department of Pathology, Faculdade de Medicina de Botucatu (FMB), Universidade Estadual Paulista (Unesp), Botucatu, São Paulo, Brazil.; II PhD. Assistant professor, Department of Microbiology and Immunology, Institute of Biosciences, Universidade Estadual Paulista (Unesp), Botucatu, São Paulo, Brazil.; III PhD. Assistant professor, Department of Biostatistics, Institute of Biosciences, Universidade Estadual Paulista (Unesp), Botucatu, São Paulo, Brazil.; IV PhD. Assistant professor, Department of Gynecology and Obstetrics, Faculdade de Medicina de Botucatu (FMB), Universidade Estadual Paulista (Unesp), Botucatu, São Paulo, Brazil.; V PhD. Assistant professor, Department of Pathology, Faculdade de Medicina de Botucatu (FMB), Universidade Estadual Paulista (Unesp), Botucatu, São Paulo, Brazil.

**Keywords:** Streptococcus agalactiae, Pregnancy, Infant, newborn, Patient isolation, Culture media, Streptococcus agalactiae, Gravidez, Recém-nascido, Isolamento de pacientes, Meios de cultura

## Abstract

**CONTEXT AND OBJECTIVE:**

Maternal *Streptococcus agalactiae* colonization and early-onset neonatal sepsis have aroused interest in the worldwide literature. Streptococcal neonatal disease is associated with significant morbidity and mortality in the perinatal period, especially among premature neonates. The aim of this study was to assess the prevalence of maternal streptococcal colonization by using combined swab cultures, compared with swab collection from a single site.

**DESIGN AND SETTING:**

Cross-sectional study at Faculdade de Medicina de Botucatu, Universidade Estadual Paulista.

**METHODS:**

Samples were obtained from 405 patients at gestational ages of 35 to 37 weeks. Swabs from the perianal (rectal) region, vaginal introitus and upper lateral vaginal vault were cultured in Todd-Hewitt selective broth. Colonies suggestive of *Streptococcus agalactiae* were subjected to the catalase and CAMP (Christie, Atkins, Munch-Petersen) tests. To evaluate the positivity of combined swab cultures, Tukey's test was used for comparison of proportions.

**RESULTS:**

The prevalence of streptococcal colonization was 25.4%. Among the patients with positive cultures, 28.1% had this at only one collection site, 24.2% simultaneously at two sites and 47.5% at all three sites. Associating the swabs from two collection sites significantly increased streptococcal isolation, compared with a single swab (P < 0.05), except for perianal (rectal) collection. Use of combined swabs from three collection sites showed statistically higher isolation rates.

**CONCLUSION:**

In combined swab cultures collected from three collection sites, the prevalence of maternal *Streptococcus agalactiae* colonization was higher than in swabs collected from a single site.

## RESUMO

**CONTEXTO E OBJETIVO:**

Colonização materna por *Streptococcus agalactiae* e sepse neonatal de início precoce têm despertado interesse na literatura mundial. A doença estreptocócica neonatal está associada com significantes índices de morbidade e mortalidade perinatal, principalmente nos recém-nascidos prematuros. O objetivo deste estudo foi avaliar a prevalência de colonização estreptocócica materna por meio da coleta de *swabs* combinados, comparada com coleta de *swab* num único local.

**TIPO DE ESTUDO E LOCAL:**

Estudo transversal na Faculdade de Medicina de Botucatu, Universidade Estadual Paulista.

**MÉTODOS:**

Foram obtidas amostras de 405 pacientes com idade gestacional entre 35 e 37 semanas. *Swabs* da região perianal retal, introito vaginal e terço distal da parede vaginal foram cultivados em caldo seletivo de Todd-Hewitt. Colônias sugestivas de *Streptococcus agalactiae* foram submetidas aos testes de catalase e CAMP (Christie, Atkins, Munch-Petersen). Para avaliação da positividade de cultura de *swabs* combinados foi empregado o teste de Tukey para comparação de proporções.

**RESULTADOS:**

A prevalência de colonização estreptocócica foi de 25,4%. Dentre as pacientes com cultura positiva, 28,1% tiveram positividade em apenas um local de coleta, 24,2% em dois locais simultaneamente e 47,5% nos três locais avaliados. Associação de *swabs* de dois locais de coleta aumentou significativamente o isolamento estreptocócico, comparado a único *swab* (P < 0,05), exceto para coleta perianal. (retal) Utilização de *swabs* combinados de três locais de coleta mostrou taxas de isolamento estatisticamente superiores.

**CONCLUSÃO:**

Na cultura de *swabs* combinados de três locais de coleta houve maior prevalência de colonização materna por *Streptococcus agalactiae*, comparada com coleta de *swabs* em um único local.

## INTRODUCTION

During the last few decades, maternal *Streptococcus agalactiae* (*S. agalactiae*) colonization and early-onset neonatal sepsis have undergone intense worldwide study. Streptococcal neonatal disease is associated with significant morbidity and mortality during the perinatal period,^1^ especially among premature neonates.^2^

Gram-positive *S. agalactiae* can colonize the genitourinary and lower gastrointestinal tract of asymptomatic women. Colonization by this organism is associated with an increased risk of urinary tract infection^3^ and pregnancy complications such as endometritis^3^ and chorioamnionitis.^3,4^ Moreover, neonates exposed to *S. agalactiae* can suffer severe consequences like pneumonia, sepsis and meningitis.^5^

Vertical transmission of *S. agalactiae* occurs when the membranes rupture and the neonate comes into contact with a colonized maternal birth canal during labor and delivery.^6^ The Centers for Disease Control and Prevention (CDC) currently recommends that all pregnant women should be screened for *S. agalactiae* at a gestational age of between 35 and 37 weeks.^7^ For patients with positive vaginal or rectal cultures, intravenous penicillin prophylaxis is recommended during labor. Implementation of this strategy has reduced early-onset neonatal sepsis by 70%.^8^

According to the literature, between 6.5%^9^ and 43.6%^10^ of pregnant women are colonized with *S. agalactiae* in the vagina or rectum. Maternal colonization rates may differ according to the culture medium and collection site.^11^ Some studies have consistently shown that combined swabs from different collection sites give up to 30% better yield than single vaginal swabs do.^12^

## OBJECTIVE

The purpose of this study was to evaluate the prevalence of maternal *S. agalactiae* colonization in combined swab cultures, compared with swabs collected from a single site.

## MATERIALS AND METHODS

This cross-sectional study was conducted at the Faculdade de Medicina de Botucatu (FMB), Universidade Estadual Paulista (Unesp) between February 2006 and January 2007. This university's hospital is located in a central area of the state of São Paulo and caters to patients of differing socioeconomic statuses. The samples were obtained from 405 women with a gestational age of between 35 and 37 weeks and singleton pregnancy who attended the FMB prenatal service.

The exclusion criteria were refusal to participate, symptoms of urinary infection over the preceding 60 days, antibiotic use over the preceding 60 days and sexual intercourse over the 72 hours preceding the examination. After the study had been approved by the ethics committee and written informed consent had been obtained from all patients, vaginal and rectal samples were collected to assess the patients’ *S. agalactiae* status.

Samples were collected using separate sterile swabs, from the vaginal introitus (VI), upper lateral vaginal vault (LV), and rectal region (RR). The samples were immediately inoculated into nonnutritive Amies transport medium for transportation to the microbiology laboratory. The swabs were then removed from the Amies medium and inoculated in Todd-Hewitt broth supplemented with colistin (10 μg/ml) and nalidixic acid (15 μg/ml). The inoculated samples were incubated for 24 hours at 37 °C. The broth was then subcultured onto a Columbia agar base with 5% sheep blood, under the same conditions. Colonies suggestive of *S. agalactiae* (because they presented a narrow zone of beta-hemolysis) were subjected to the catalase and CAMP tests. Negative blood-agar plates were reincubated for an additional 18 to 24 hours and reexamined.

Statistical analyses were performed using the SigmaStat software, version 3.1. Comparisons were performed using the Tukey test with significance set at P < 0.05.

## RESULTS

During the study period, 567 patients with a gestational age of 35 to 37 weeks were seen at the FMB prenatal service. A total of 162 patients were excluded: 106 because they had been taking antibiotics and 56 because they refused to participate in the study. Considering all the 405 women included in the study, the results from the cultures showed that the rate of maternal colonization with *S. agalactiae* was 25.4%.

With regard to the collection site, the prevalence of *S. agalactiae* detection was evaluated by means of single and combined swabs from VI, LV and RR. A total of 103 culture-positive samples were included in this analysis. Out of all of the women with *S. agalactiae* colonization, 14.5% presented positive cultures both from VI and from LV; 5.8% from VI and RR, and 3.9% from LV and RR. Positive cultures from all three collection sites were found in 47.5%, and from only one site in 28.1% (9.7% from VI; 3.9% from LV and 14.5% from RR).

Statistical analysis on the effectiveness of associating swabs from different sites revealed that using a combination of two swabs increased the yield of *S. agalactiae* in relation to using a single swab, except when the swab was obtained from the rectal region. As shown in Table 1, association of swabs from the three collection sites significantly increased the detection of maternal *S. agalactiae* colonization.

**Table 1.** Number of *Streptococcus agalactiae* isolates according to collection site

	*Streptococcus agalactiae* isolates	
	n (%)	Combined swabs n (%)
VI	10 (9.7)^c^	-
LV	4 (3.9)^c^	-
RR	15 (14.5)^bc^	-
VI + LV	5 (14.5)^b^	29 (28.1)^b^
VI + RR	6 (5.8)^b^	31 (30.1)^b^
LV + RR	4 (3.9)^b^	23 (22.3)^b^
VI + LV + RR	49 (47.5)^a^	103 (100.0)^a^

Tukey test. Small letters used for group comparisons; values followed by same letter do not differ. VI = vaginal introitus; LV = upper lateral vaginal vault; RR = rectal region; VI + LV = combined vaginal introitus and upper lateral vaginal vault; VI + RR = combined vaginal introitus and rectal region; LV + RR = combined upper lateral vaginal vault and rectal region; VI + LV + RR = combined vaginal introitus, upper lateral vaginal vault and rectal region. Small letters used for group comparisons; values followed by same letter do not differ.

## DISCUSSION

The prevalence of maternal colonization with *S. agalactiae* among the 405 women included in this study was similar to reports in the literature.^13-15^ Given that the culture sensitivity may vary according to the anatomical collection site and microbiological methods, the procedures used in this study to detect *S. agalactiae* were in accordance with the CDC recommendations.^12^

With regard to evaluating combined swabs from vaginal sites, vaginal samples (from the introitus and lateral vault) were able to isolate more *S. agalactiae* than were rectal samples. These results are in agreement with findings from other studies, which also found higher detection rates in vaginal swabs than in rectal swabs.^16,17^

Considering the effect of each anatomical site on *S. agalactiae* detection, the rectal region showed higher isolation frequency. According to Platt et al.,^18^ there is a significant increase in the streptococcal isolation rate when the rectal region is also assessed than when just using vaginal cultures. One study conducted in northeastern Brazil^19^ showed that 22% of the pregnant women colonized by *S. agalactiae* would have had negative culture results if the rectal region had not been evaluated. Because the gastrointestinal tract is a natural reservoir for *S. agalactiae* and it is the likely source of vaginal colonization, culturing of rectal swabs is of great importance in assessing the streptococcal status.

Our findings agree with the literature^19-21^ that swabbing from two or three anatomical sites significantly increases the detection rate in relation to swabbing from one site. Furthermore, the use of three collection sites (vaginal introitus, lateral vaginal vault and rectal region) gave statistically superior *S. agalactiae* detection rates.

## CONCLUSION

In combined swab cultures collected from three different sites, the prevalence of maternal *Streptococcus agalactiae* colonization was higher than the prevalence observed in swabs collected from a single site.
